# A study of linear measurement and clinical correlation of brain atrophy in Wilson's disease

**DOI:** 10.3389/fnhum.2023.1142082

**Published:** 2023-03-28

**Authors:** Yun Wang, Hongxia Xuan, Tun Zhao, Xiaodong Li, Shujuan Li, Wenli Hu

**Affiliations:** ^1^Department of Neurology, Beijing Chaoyang Hospital, Capital Medical University, Beijing, China; ^2^Department of Neurology, Hongda Hospital, Jiamusi University, Jiamusi, China

**Keywords:** Wilson's disease, brain atrophy, UWDRS, linear measurement, clinical correlation

## Abstract

**Background:**

The aim of this study was to explore the clinical relevance of linear measures of Wilson's disease (WD).

**Methods:**

Relative values of brain atrophy in 30 patients with WD and 30 healthy volunteers were measured and compared using a manual measurement method. Linear measurement indicators of brain atrophy in patients with and without mental disorders were also compared. In addition, correlations of patients' age, disease duration, and Unified Wilson's Disease Rating Scale (UWDRS) scores with brain atrophy indicators were determined.

**Results:**

The results showed that the e-value, Huckman number, Evans index, and lateral ventricular body index were higher in the WD group compared with the control group. The age of patients with WD was negatively correlated with the *k*-value and significantly positively correlated with the brainstem index. WD duration was prominently positively correlated with the d-value and negatively correlated with the *j*-value. In addition, neurological function scores were significantly positively correlated with the c-value, *e*-value, caudate nucleus index, Huckman number, Evans index, and lateral ventricular body index. By contrast, patients with psychiatric symptoms had a higher a-value and fourth ventricular index than those without psychiatric symptoms.

**Conclusion:**

Therefore, it can be concluded that patients with WD and those with psychiatric symptoms have more severe brain atrophy compared to normal subjects. The patient's age, disease duration, and neurological function scores were positively correlated with the severity of brain atrophy.

## 1. Introduction

Wilson's disease (WD) is an autosomal recessive disorder that occurs more frequently in adolescence (Fernando et al., 2020). It is chronic and progressive condition that can trigger abnormal copper metabolism in multiple organs of the body, resulting in a series of diseases due to systemic copper accumulation (Kitzberger et al., 2005), even with lifelong medication (Hedera, 2019). Previous studies indicated that WD has various initial symptoms (Pfeiffer, 2016) and a high misdiagnosis rate at an early stage. With complicated and diverse clinical manifestations, it may present with a single system or multisystem involvement and can be classified into the brain type, liver type, and mixed type (Poujois et al., 2017; Guindi, 2019; Mulligan and Bronstein, 2020), of which brain damage provoked by WD has brought great distress to people's life and health.

Brain atrophy caused by WD is very common, occurring in 31.6% of patients (Zhong et al., 2019; Du and Bydder, 2021), and is associated with copper accumulation in the brain (Czlonkowska et al., 2018; Dusek et al., 2019). One study found a correlation with WD nerve injury by measuring the longitudinal brain atrophy rate (Smolinski et al., 2022). Moreover, brain atrophy is a marker of increased risk of death (Lauksio et al., 2021; van den Berg, 2022), which can be divided into cerebral atrophy, cerebellar atrophy, and brain stem atrophy (Hayashi et al., 2008; Christova et al., 2017; Gellersen et al., 2017). As a chronic disease, brain atrophy leads to memory loss, decreased concentration, and language disorders (Park and Reuter-Lorenz, 2009; Callisaya et al., 2019; Mungas et al., 2021). Importantly, it is not easily detected owing to its slow development, but once the condition worsens, it will seriously affect the health and life of patients. Therefore, early detection and timely treatment can minimize the impact of brain atrophy on patients and bring the best results (Sastre-Garriga et al., 2020). Previous studies have shown that neuroimaging (Shribman et al., 2022), sNfL levels (Wang et al., 2022), the Unified Wilson's Disease Rating Scale (UWDRS) neurological and psychiatric subscales, and the GAS of the WD Tier 2 score (Volpert et al., 2017) can be used as biomarkers of WD (Pfeiffer, 2020). Currently, MRI has been reported to be mostly used to diagnose brain atrophy and classify it into diffuse brain atrophy and localized cerebral atrophy based on its scope, degree, and location (Planche et al., 2021). In addition, the third ventricle width, fourth ventricular index, frontal angle index, caudate nucleus index, Evans index, Huckman number, lateral ventricular body index, brainstem index, superior cerebellar cistern index, and sulcus width are usually determined using visual and linear measurements. The Huckman number is the sum of the widths of the anterior and posterior ventricular horns on both sides (Chrzan et al., 2019), the Evans index is the ratio of the maximum distance between the bilateral anterior ventricular horns in the axial position and the maximum internal diameter of the cranial cavity at the same level, and an Evans index value of >0.3 indicates ventricular enlargement (Chrzan et al., 2019; Zhou and Xia, 2021). However, the association between the severity and clinical features of brain atrophy in patients with WD remains unclear.

This study aimed to compare the linear measurement indicators of brain atrophy in patients with WD and healthy controls and quantitatively analyze the atrophy of different brain structures in patients with WD to investigate the correlation between the degree of brain atrophy and clinical manifestations.

## 2. Methods

### 2.1. Research object

A total of 30 patients with the brain type of WD, 15 men and 15 women, aged 15–54 (31.60 ± 11.03) years, with disease duration ranging from 0.5 to 24 years, diagnosed in the Neurology Department of the Beijing Chaoyang Hospital from January 2017 to January 2022, were involved in this study according to the diagnosed criteria of the Chinese Guidelines for Diagnosis and Treatment of Hepatolenticular Degeneration 2021 (Li, 2022). All patients have neurological and/or psychiatric symptoms. Among them, 20 (20/30, 66.67%) had limb tremors, 18 (18/30, 60.00%) had rigidity, and nine (9/30, 30.00%) had memory loss and decreased concentration. Meanwhile, 11 (11/30, 36.67%) had different degrees of psychiatric symptoms, including insomnia, anxiety, depression, visual hallucinations, auditory hallucination, and suicide impulsion, and 27 (27/30, 90.00%) had KF rings. In addition, 18 (18/30, 60.00%) had hepatic dysfunction and eight (8/30, 26.67%) had gastrointestinal symptoms such as constipation. Among the female patients, four (4/15, 26.67%) had irregular menstruation and five (5/15, 33.33%) had a history of abortion. All patients were on medication. Except for two cases that took zinc only, other patients were treated with penicillium or penicillium combined with zinc.

Thirty healthy volunteers without underlying diseases and neurological symptoms, aged 16–54 (31.30 ± 10.40) years, were recruited as controls, including 14 men and 16 women. All participants had signed informed consent. The study was approved by the Ethics Committee of the Beijing Chaoyang Hospital, Capital Medical University (2022-KE-484).

### 2.2. Clinical evaluation

All patients with WD were examined using the UWDRS score (Volpert et al., 2017). The full UWDRS consists of three subscales: the neurological subscale, the hepatic subscale, and the psychiatric subscale. It represents the three main features of the clinical presentation of WD.

### 2.3. Linear measurement of brain atrophy

Head MRI was performed on all patients and normal controls. Brain imaging was carried out by the same neurologist, and the required diameters of each case were measured two times and then recorded and averaged.

The measured indicators were as follows: (1) a, the maximum transverse diameter of the fourth ventricular; h, the maximum transverse diameter of the posterior cranial fossa brain tissue; and a/h, the fourth ventricular index; (2) b, the maximum distance between the frontal horns of bilateral brain ventricles; i, the transverse diameter of the brain tissue at the b level; and b/i, the frontal angle index; (3) c, the minimum distance between the two caudate nuclei of frontal horns of bilateral brain ventricles; j, the transverse diameter of the brain tissue at the c level; and c/j, the caudate nucleus index; (4) b + c, the Huckman number and c/b, the Evans index; (5) d, the width of the third ventricle; (6) e, the minimum distance between the lateral walls of bilateral brain ventricles; k, the brain tissue at the e level and e/k, the lateral ventricular body index; (7) f, pontine cistern width; n, the distance from the median point of the dorsum sellae posterior border to the fourth ventricle anterior border; and f/n, the brainstem index; (8) g, the width of the anterior part of the superior cerebellar cistern; o, the brain tissue at the g level; g/o, the superior cerebellar cistern index; and (9) taking the average value obtained by dividing the sum of the four widest sulcus values at the highest scan level by 4 as the measured value of sulcus 1, and a value of > 0.5 cm was considered as sulcus enlargement.

Transverse diameters of the cerebral ventricular system were represented by a, b, c, d, e, f, and g. Their values were positively related to the degree of brain atrophy, and the greater the transverse diameters, the more serious the brain atrophy. In addition, transverse diameters of the brain tissue were denoted by h, i, j, k, n, and o. The ratio of the transverse diameter of the ventricles to the corresponding transverse diameter of the brain tissue was known as the measured relative value of brain atrophy, and the greater the relative value, the more severe the brain atrophy.

### 2.4. MRI acquisition

MRI was performed with a 3.0-T scanner (Trio, Siemens) with a 20-channel birdcage head coil. The MRI scanning sequences included T1-weighted imaging [sagittal slice thickness, 5.0 mm; slice spacing, 0.35 mm; repetition time msec/echo time ms, 2,000/9; field of view, 230 × 230 mm; spatial resolution: 1.2 × 1.2 × 5 mm], T2-weighted imaging [sagittal slice thickness, 5.0 mm; slice spacing, 0.35 mm; repetition time ms/echo time ms, 5,000/94; field of view, 230 × 230 mm; spatial resolution: 1.2 × 1.2 × 5 mm], diffusion-weighted imaging (DWI) [sagittal slice thickness, 5.0 mm; slice spacing, 0.35 mm; repetition time ms/echo time ms, 4,000/86; field of view, 230 × 230 mm, spatial resolution: 1.2 × 1.2 × 5 mm], and liquid flip recovery attenuation sequence (FLAIR) [sagittal slice thickness, 5.0 mm; slice spacing, 0.4 mm; repetition time ms/echo time ms, 2,000/9; field of view, 190 × 190 mm, spatial resolution: 1.2 × 1.2 × 5 mm]. The MRI protocol also included sagittal T1-weighted three-dimensional fast spin-echo acquisition, with the following parameters: sagittal slice thickness, 5.0 mm; slice spacing, 0.4 mm; repetition time ms/echo time ms, 2,000/9; field of view, 190 × 190 mm; and spatial resolution: 1.2 × 1.2 × 5 mm.

### 2.5. Statistical analysis

Statistical analysis was conducted using SPSS version 22.0. Continuous data in normal distribution were expressed as mean ± standard deviation, and Student's *t-test* was applied for comparison between the two groups; continuous data not in normal distribution were presented as M (Q1–Q3), and the Mann–Whitney U-test was carried out for comparison between the two groups. For bivariate normally distributed, Pearson's analysis was performed, and for bivariate with at least one variable did not normally distribute, Spearman's analysis was employed. A *P* < 0.05 was considered a significant difference.

## 3. Results

### 3.1. UWDRS score of patients with WD

As shown in Table 1, the average scores of the UWDRS, neurological function, psychiatric symptom, and liver function of the 30 patients were 37.50 (9.25–76.25), 29.00 (7.00–66.25), 2.00 (0–7.00), and 0.50 (0–2.00), respectively.

**Table 1:** Basic information of 30 patients with WD.

**Gender**	**Age (years)**	**Disease duration (years)**	**Neurological function score**	**Psychiatric function score**	**Liver function score**
Female	19	5	82	0	1
Female	30	4	67	7	0
Female	27	21	0	0	0
Male	24	3	5	0	0
Female	55	5	24	7	0
Male	23	4	7	0	0
Male	36	20	102	5	4
Male	39	5	35	0	12
Male	45	4	7	7	0
Male	34	1	6	1	1
Female	16	13	8	5	0
Male	54	22	64	9	4
Male	27	12	74	16	2
Female	19	11	99	37	2
Male	29	4	109	26	0
Male	21	7	14	1	1
Male	39	12	17	0	0
Female	34	13	27	0	0
Male	31	4	52	10	2
Female	40	4	0	0	1
Female	33	11	21	3	2
Female	24	18	0	0	0
Male	31	17	7	0	0
Female	41	24	90	2	0
Female	53	18	44	3	0
Female	52	8	39	2	5
Male	20	1	108	34	4
Female	35	0.5	31	12	1
Female	42	3	57	1	0
Male	40	1	0	2	2

### 3.2. Comparison of linear measurement indicators of brain atrophy between patients with WD and healthy controls

As shown in Table 2, the WD group had a lower h-value (*P* < 0.001) and k-value (*P* = 0.005) and a higher e-value (*P* = 0.001) compared to the control group. In addition, the Huckman number (*P* < 0.001), Evans index (*P* < 0.001), and lateral ventricular body index (*P* < 0.001) in the WD group were higher than those in the control group. Therefore, patients with WD had more serious brain atrophy.

**Table 2:** Linear measurement indicators of brain atrophy in WD patients and healthy controls.

	**WD group**		**Control group**		***P*-value**
	**Mean value**	**SD**	**Mean value**	**SD**	
a	13.99	3.68	12.87	1.80	0.138
h	101.10	6.42	107.25	5.26	<0.001
n	30.44	5.75	32.53	3.21	0.088
f	9.37	1.93	7.40	1.34	<0.001
b	32.18	2.34	32.77	1.75	0.275
i	105.57	3.43	105.94	18.35	0.912
c	16.39	4.96	11.15	2.43	<0.001
j	115.39	4.80	111.54	27.63	0.456
d	10.67	2.64	10.12	1.22	0.301
e	22.92	5.34	18.73	3.20	0.001
k	120.39	5.92	124.73	5.49	0.005
g	21.91	3.34	21.00	4.18	0.358
o	126.04	21.67	131.82	23.22	0.323
a/h	0.14	0.04	0.12	0.02	0.018
b/i	0.30	0.02	0.39	0.52	0.350
c/j	0.14	0.04	0.15	0.22	0.838
b+c	48.57	5.74	43.92	3.55	<0.001
c/b	0.51	0.16	0.34	0.07	<0.001
e/k	0.19	0.04	0.15	0.02	<0.001
f/n	0.40	0.60	0.23	0.04	0.114
g/o	0.21	0.21	0.20	0.26	0.874
Brain sulcus measurements	4.65	0.61	4.65	0.61	0.987

a, the maximum transverse diameter of the fourth ventricle; h, the maximum transverse diameter of the fourth ventricular tissue; n, distance from median point of dorsum sellae posterior border to the fourth ventricle anterior border; f, pontine cistern width; b, the maximum distance between the frontal horns of bilateral brain ventricles; i, the transverse diameter of brain tissue at b level; c, the minimum distance between the two caudate nucleus of frontal horns of bilateral brain ventricles; j, the transverse diameter of brain tissue at c level; d, third ventricle width; e, the minimum distance between the lateral walls of bilateral brain ventricles; k, brain tissue at e level; g, the width of the anterior part of superior cerebellar cistern; o, brain tissue at the level of the superior cerebellar cistern; a/h, the fourth ventricular index; b/i, frontal angle index; c/j, caudate nucleus index; b+c, Huckman's value; c/b, Evans index; e/k, lateral ventricular body index; f/n, brainstem index; g/o, superior cerebellar cistern index.

### 3.3. Correlation between age, disease duration, and linear measurement indicators of brain atrophy in patients with WD

As presented in Table 3, the age of patients with WD was dramatically negatively correlated with the *k*-value (*P* = 0.047, r = −0.365) and significantly positively correlated with the brainstem index (*P* = 0.049, r = 0.362). Furthermore, most of the ventricular system transverse diameters and the measured relative values of brain atrophy were positively correlated with the age of patients. Although there was no statistically significant difference, it had a certain reference value. These findings indicated that older patients with WD had worse brain atrophy. In addition, we found that the disease duration of patients with WD was markedly positively correlated with the d-value (*P* = 0.027, r = 0.403) but prominently negatively related to the j-value (*P* = 0.027, r = −0.403). Most of the measured relative values of brain atrophy had a positive link to the age of patients. However, no statistically significant difference was detected. Thus, it could be seen that WD patients with longer disease duration had more serious brain atrophy.

**Table 3:** Correlation between age, disease duration, and linear measurement indicators of brain atrophy in WD patients.

	**Correlation between disease duration and brain atrophy indicators**		**Correlation between the age of WD patients and brain atrophy indicators**	
	*P* **-value**	**R-value**	*P* **-value**	**R-value**
a	0.571	−0.108	0.286	−0.201
h	0.400	−0.159	0.507	−0.126
n	0.140	−0.276	0.270	−0.208
f	0.981	−0.004	0.087	0.318
b	0.068	0.337	0.140	0.276
i	0.082	0.322	0.733	−0.065
c	0.683	−0.078	0.872	0.031
j	0.027	−0.403	0.124	−0.287
d	0.027	0.403	0.815	−0.045
e	0.358	−0.174	0.699	0.074
k	0.316	0.189	0.047	−0.365
g	0.204	0.239	0.662	0.083
o	0.360	−0.173	0.456	0.141
a/h	0.737	−0.064	0.345	−0.179
b/i	0.237	0.223	0.060	0.347
c/j	0.490	−0.131	0.753	0.060
b+c	0.712	0.070	0.464	0.139
c/b	0.356	−0.175	0.812	−0.045
e/k	0.271	−0.208	0.422	0.152
f/n	0.079	0.326	0.049	0.362
g/o	0.200	0.241	0.435	−0.148
Brain sulcus measurements	0.316	0.190	0.282	0.203

a, the maximum transverse diameter of the fourth ventricle; h, the maximum transverse diameter of the fourth ventricular tissue; n, distance from median point of dorsum sellae posterior border to the fourth ventricle anterior border; f, pontine cistern width; b, the maximum distance between the frontal horns of bilateral brain ventricles; i, the transverse diameter of brain tissue at b level; c, the minimum distance between the two caudate nucleus of frontal horns of bilateral brain ventricles; j, the transverse diameter of brain tissue at c level; d, third ventricle width; e, the minimum distance between the lateral walls of bilateral brain ventricles; k, brain tissue at e level; g, the width of the anterior part of superior cerebellar cistern; o, brain tissue at the level of the superior cerebellar cistern; a/h, the fourth ventricular index; b/i, frontal angle index; c/j, caudate nucleus index; b+c, Huckman's value; c/b, Evans index; e/k, lateral ventricular body index; f/n, brainstem index; g/o, superior cerebellar cistern index.

### 3.4. Correlation between the UWDRS neurological function score and the linear measurement indicators of brain atrophy in patients with WD

In the present study, we observed that the neurological function score was notably positively related to the c-value (*P* = 0.001, r = 0.597) and the e-value (*P* < 0.001, r = 0.706) (Table 4). In addition, it was significantly positively correlated with many measured relative values of brain atrophy, including caudate nucleus index (*P* = 0.001, r = 0.596), Huckman number (*P* = 0.002, r = 0.535), Evans index (*P* = 0.001, r = 0.583), and lateral ventricular body index (*P* < 0.001, r = 0.676). These results suggested that the higher the neurological function score, the more severe the brain atrophy.

**Table 4:** Correlation between the UWDRS neurological function score and the linear measurement indicators of brain atrophy in WD patients.

	**Neurological function score**	
	*P* **-value**	**R-value**
a	0.102	0.305
h	0.341	−0.180
n	0.554	−0.112
f	0.529	0.120
b	0.803	0.048
i	0.784	−0.052
c	0.001	0.597
j	0.918	0.020
d	0.580	0.105
e	<0.001	0.706
k	0.332	0.184
g	0.640	−0.089
o	0.273	0.207
a/h	0.051	0.360
b/i	0.677	0.079
c/j	0.001	0.596
b+c	0.002	0.535
c/b	0.001	0.583
e/k	<0.001	0.676
f/n	0.482	0.133
g/o	0.204	−0.239
Brain sulcus measurements	0.259	0.213

a, the maximum transverse diameter of the fourth ventricle; h, the maximum transverse diameter of the fourth ventricular tissue; n, distance from median point of dorsum sellae posterior border to the fourth ventricle anterior border; f, pontine cistern width; b, the maximum distance between the frontal horns of bilateral brain ventricles; i, the transverse diameter of brain tissue at b level; c, the minimum distance between the two caudate nucleus of frontal horns of bilateral brain ventricles; j, the transverse diameter of brain tissue at c level; d, third ventricle width; e, the minimum distance between the lateral walls of bilateral brain ventricles; k, brain tissue at e level; g, the width of the anterior part of superior cerebellar cistern; o, brain tissue at the level of the superior cerebellar cistern; a/h, the fourth ventricular index; b/i, frontal angle index; c/j, caudate nucleus index; b+c, Huckman's value; c/b, Evans index; e/k, lateral ventricular body index; f/n, brainstem index; g/o, superior cerebellar cistern index.

### 3.5. Comparison of linear measurement indicators of brain atrophy between patients with and without psychiatric symptoms

As shown in Table 5, patients with psychiatric symptoms exhibited a higher a-value (*P* = 0.013) and a fourth ventricular index (*P* = 0.006) than those without psychiatric symptoms. Moreover, most measured relative values of brain atrophy were higher in patients with psychiatric symptoms than in those without psychiatric symptoms. Therefore, brain atrophy in patients with psychiatric symptoms was more severe.

**Table 5:** Linear measures of brain atrophy between WD patients with and without psychiatric symptoms.

	**With psychiatric symptoms**		**Without psychiatric symptoms**		
	**Mean value**	**SD**	**Mean value**	**SD**	*P* **-value**
a	16.11	3.09	12.76	3.48	0.013
h	100.21	4.41	101.62	7.41	0.570
n	28.35	8.66	31.66	2.73	0.131
f	9.06	2.26	9.55	1.75	0.515
b	31.45	2.47	32.61	2.22	0.197
i	105.33	2.47	105.70	3.94	0.784
c	17.42	6.26	15.78	4.10	0.393
j	114.81	5.20	115.72	4.67	0.624
d	11.55	3.31	10.16	2.09	0.169
e	24.46	6.62	22.03	4.39	0.235
k	119.69	5.68	120.79	6.17	0.632
g	22.03	4.45	21.83	2.64	0.877
o	129.88	3.75	123.82	27.10	0.470
a/h	0.16	0.03	0.13	0.03	0.006
b/i	0.30	0.02	0.31	0.02	0.175
c/j	0.15	0.05	0.14	0.04	0.367
b+c	48.87	7.23	48.39	4.89	0.830
c/b	0.55	0.20	0.49	0.13	0.266
e/k	0.20	0.05	0.18	0.04	0.211
f/n	0.58	0.99	0.30	0.05	0.222
g/o	0.17	0.03	0.24	0.26	0.424
Brain sulcus measurements	4.91	0.80	4.50	0.41	0.071

a, the maximum transverse diameter of the fourth ventricle; h, the maximum transverse diameter of the fourth ventricular tissue; n, distance from median point of dorsum sellae posterior border to the fourth ventricle anterior border; f, pontine cistern width; b, the maximum distance between the frontal horns of bilateral brain ventricles; i, the transverse diameter of brain tissue at b level; c, the minimum distance between the two caudate nucleus of frontal horns of bilateral brain ventricles; j, the transverse diameter of brain tissue at c level; d, third ventricle width; e, the minimum distance between the lateral walls of bilateral brain ventricles; k, brain tissue at e level; g, the width of the anterior part of superior cerebellar cistern; o, brain tissue at the level of the superior cerebellar cistern; a/h, the fourth ventricular index; b/i, frontal angle index; c/j, caudate nucleus index; b+c, Huckman's value; c/b, Evans index; e/k, lateral ventricular body index; f/n, brainstem index; g/o, superior cerebellar cistern index.

## 4. Discussion

Wilson's disease is an autosomal recessive inherited disease caused by gene mutation and is often misdiagnosed (Lorincz, 2010). Brain atrophy was most evident in the central structure of patients with WD (Dusek et al., 2021). Du and Bydder (2021) suggested that brain atrophy is a good biomarker for evaluating the clinical severity of WD. In this study, the degree of brain atrophy in patients was analyzed using linear measurement, and the association of brain atrophy severity with the clinical manifestations in patients was further explored. Our results revealed that patients with WD had more serious brain atrophy than healthy individuals, and the age, disease duration, and neurological function score were all positively correlated with the severity of brain atrophy. In addition, patients with psychiatric symptoms showed more serious brain atrophy.

This study compared WD patients with healthy controls based on linear measurement indicators after the head MRI, attempting to investigate the brains of patients. MRI is an important tool for detecting brain atrophy and increasing research is trying to explore and expand its functions (Dusek et al., 2020; Shribman et al., 2021). Studies suggested that abnormalities in the putamen nucleus, the pons, the midbrain, and the thalamus are commonly found in WD *via* MRI (Yu et al., 2019). Our study indicated that the WD group had a higher Huckman number (*P* < 0.001), Evans index (*P* < 0.001), and lateral ventricular body index (*P* < 0.001). The study by Hamed et al. revealed a correlation between age and brain atrophy (Hamed et al., 2020). However, the association of patients' age and disease duration with the linear measurement indicators of brain atrophy caused by WD is yet to be adequately studied. In this study, we observed that the age of patients was markedly positively linked to the brainstem index (*P* = 0.049, r = 0.362). In addition, the disease duration was positively correlated with the degree of brain atrophy.

The UWDRS plays an essential role in assessing the severity of WD (Leinweber et al., 2008), and it has also been demonstrated to be associated with WD-induced brain atrophy (Dusek et al., 2021). Our study found that the neurological function score, psychiatric symptom score, and liver function score were low in patients with WD, and the correlation between neurological function score and linear measurement indicators of brain atrophy caused by WD remained to be investigated. Smolinski et al. found that patients with neurological manifestations had significantly higher annualized atrophy rates than other patients (Smolinski et al., 2022) and that the volume of all major brain structures correlated with functional and neurological impairment in patients with WD (Smolinski et al., 2019). In line with previous studies which revealed an association between brain atrophy and cognitive functions (Nagai et al., 2008; Liu et al., 2016), Kalita et al. found that midbrain atrophy was associated with nervous system severity through 3D BRAVO sequence measurements of the midbrain and the pons (Kalita et al., 2017). Song et al. quantified brain morphological abnormalities by whole brain volume and cortical thickness using 3D T1-weighted images (Song et al., 2021). We observed that the neurological function score was significantly positively correlated with various measured relative values of brain atrophy. Furthermore, as psychiatric dysfunctions are crucial clinical features of patients with WD (Pfeiffer, 2016), the present study compared the linear measurement indicators of brain atrophy in patients with and without psychiatric symptoms, indicating that patients with psychiatric symptoms had a higher a value (*P* = 0.013) and fourth ventricular index (*P* = 0.006).

In conclusion, by exploring the relationship between the linear measurement indicators of WD-caused brain atrophy and the clinical manifestations, we found that patients with WD have more serious brain atrophy than healthy individuals and those with longer disease duration and older age exhibit worse brain atrophy. The results also suggest that the neurological function scores of patients with WD were positively correlated with the severity of brain atrophy. In addition, patients with psychiatric symptoms have more severe brain atrophy. However, this study also has some limitations. The number of participants in our study was limited; thus, the correlation between the degree of brain atrophy and clinical manifestations in patients with WD still needs to be further studied by analyzing more clinical features and a larger number of subjects. In addition, in future research studies, we will try to use more sophisticated measures of brain atrophy and explore the relations between the measures and the pathological changes of WD, such as the damage to specific deep gray matter nuclei. Since brain atrophy caused by WD is a pressing problem, more explorations and research studies are needed to resolve it.

## Data availability statement

The raw data supporting the conclusions of this article will be made available by the authors, without undue reservation.

## Ethics statement

The study was approved by the Ethics Committee of Beijing Chaoyang Hospital, Capital Medical University (2022-KE-484). The patients/participants provided their written informed consent to participate in this study.

## Author contributions

YW and HX designed and conceived this study. TZ and XL analyzed the data and created the tables. SL and WH contributed to the measurement of MRI images and wrote the article. All authors reviewed and approved the manuscript.
